# HJURP modulates cell proliferation and chemoresistance via the MYC/TOP2A transcriptional axis in gastric cancer

**DOI:** 10.3389/fmolb.2025.1566293

**Published:** 2025-04-11

**Authors:** Xu Li, Xiwen Li, Yanlin Ren, Ling Wang, Zehao Mao, Shikun Gao, Peng Ma, Junjie Chen

**Affiliations:** ^1^ Department of Gastrointestinal Surgery, Affiliated Hospital of Nantong University, Medical School of Nantong University, Nantong, China; ^2^ Clinical Medical Research Center, Affiliated Hospital of Nantong University, Nantong, China; ^3^ Department of Central Laboratory, Kunshan Hospital of Chinese Medicine, Affiliated Hospital of Yangzhou University, Kunshan, China; ^4^ Department of Labor Hygiene and Occupational Disease Prevention and Control, Nantong Center for Disease Control and Prevention, Nantong, China; ^5^ Department of Hematology, Affiliated Hospital 2 of Nantong University, Nantong, China; ^6^ Nantong Key Laboratory of Gastrointestinal Oncology, Affiliated Hospital of Nantong University, Nantong, China

**Keywords:** gastric cancer, HJURP, MYC/TOP2A, proliferation, chemoresistance

## Abstract

**Background:**

The histone chaperone Holliday Junction Recognition Protein (HJURP) has been associated with multiple types of cancers, but its role in GC is not yet fully understood. Considering its functions in centromere stability and DNA repair, investigating HJURP’s role in GC may offer novel therapeutic perspectives.

**Methods:**

HJURP expression was examined in a dataset comprising TCGA-STAD samples and an internal group of GC patients, utilizing RNA sequencing and Western blot techniques. Functional experiments were carried out on the AGS and HGC-27 GC cell lines. The expression levels of HJURP, MYC, and Topoisomerase II alpha (TOP2A) were assessed via quantitative real-time PCR and Western blot. Proliferation rates of the cells were determined through EdU, CCK-8, and colony formation assays.

**Results:**

Compared to adjacent normal tissues, HJURP expression was notably increased in GC tissues, a finding consistent across both the TCGA-STAD database and our internal patient group. Silencing HJURP markedly reduced GC cell growth and chemoresistance. Mechanistically, HJURP enhanced MYC stability, which in turn promoted TOP2A transcription. Rescue experiments confirmed that overexpression of TOP2A alters proliferation and chemoresistance of GC cells with HJURP knockdown, indicating the dependency of this axis on MYC activity.

**Conclusion:**

Our study demonstrates that HJURP is critical for promoting GC proliferation and chemoresistance through the regulation of the MYC/TOP2A transcriptional network. Targeting HJURP might offer a novel therapeutic avenue for GC, necessitating further exploration of its clinical potential. This work underscores the value of investigating histone chaperones as potential targets in cancer treatment.

## 1 Introduction

Gastric cancer (GC) is a particularly aggressive and deadly cancer that is difficult to detect early due to its vague symptoms, frequently leading to diagnosis at advanced stages and a 5-year survival rate of less than 20% (Chen et al., 2020; Siegel et al., 2024). The dietary habits, *Helicobacter pylori* infection and environmental factors contribute significantly to the incidence rates (Machlowska et al., 2020; Ou et al., 2024a). Despite advancements in surgical techniques, chemotherapy, and targeted therapies, the prognosis remains grim, particularly when the disease is not caught early. Furthermore, both inherent and acquired resistance to therapy are significant barriers to successful treatment (Lu and Zhang, 2022; He et al., 2024). Hence, there is an urgent requirement to develop innovative strategies and identify more effective early diagnostic markers to enhance the prognosis for GC patients.

The Holliday Junction Recognition Protein (HJURP, also referred to as hFLEG1) is a key centromeric protein involved in the incorporation and maintenance of the histone H3-like variant CENP-A at centromeres (Serafim et al., 2024). Across multiple carcinomas, HJURP has exhibited upregulation and a strong correlation with patient survival outcomes (Li et al., 2023; Jia et al., 2024). It has been identified as a prognostic biomarker and potential therapeutic target in cancers such as oral cancer (Tsevegjav et al., 2022) and clear cell renal cell carcinoma (Zhang F. et al., 2021). NFE2L1 mitigates ferroptosis by transcriptionally regulating HJURP, playing a role in the development of oral squamous cell carcinoma (Zhang et al., 2023). In ovarian cancer, HJURP inhibits cell proliferation by influencing CENP-A/CENP-N interactions (Zhang et al., 2022), while in prostate cancer, it enhances cell proliferation by increasing CDKN1A degradation through the GSK3β/JNK signaling pathway (Lai et al., 2021). A comprehensive analysis in hepatocellular carcinoma revealed that ASF1A and HJURP serve as a two-gene prognostic model due to their involvement in H3-H4 histone chaperone functions (Liu Y. et al., 2024). HJURP appears to play a crucial role in modulating chemoradiotherapy resistance in GC. In triple-negative breast cancer, it regulates chemoresistance via the YAP1/NDRG1 transcriptional axis (Mao et al., 2022). Moreover, HJURP aids in DNA repair by facilitating chromatin reorganization at double-strand break sites in astrocytoma (Serafim et al., 2024). Reducing HJURP levels disrupts clonogenic capacity and increases radiation-induced cell death in glioblastoma cells (Serafim et al., 2020). However, the expression patterns, functional roles, and prognostic implications of HJURP in GC remain largely unknown and warrant further investigation.

Topoisomerase II alpha (TOP2A), an enzyme critical for DNA replication and cell division, has attracted considerable attention in cancer research due to its pivotal role in tumor development and its potential as a therapeutic target (Uuskula-Reimand and Wilson, 2022; Liu et al., 2023; Zhang J. et al., 2025; Zou et al., 2025). This nuclear enzyme plays key roles in processes like chromosome condensation, chromatid segregation, and relieving torsional stress during DNA transcription and replication (Zhang et al., 2020). TOP2A facilitates these processes by temporarily breaking and rejoining double-stranded DNA, enabling the strands to pass through one another and modifying DNA topology. Two forms of this enzyme are believed to have arisen from a gene duplication event. The gene for the alpha form is situated on chromosome 17, whereas the gene for the beta form resides on chromosome 3. Research indicates that aberrant expression or amplification of TOP2A is common in various malignancies, including breast, lung, and GC, correlating with poor prognosis and resistance to chemotherapy (Li et al., 2022; Lee et al., 2023). Current studies are focused on elucidating the mechanisms underlying TOP2A’s involvement in tumor progression and the development of resistance to treatment. Moreover, there is a growing interest in integrating TOP2A expression analysis into personalized medicine strategies, aiming to predict response to anthracycline-based chemotherapy, a treatment modality that targets TOP2A. Advances in genomics have facilitated the identification of co-amplified genes within the TOP2A amplicon, providing insights into potential synergistic targets for combinatorial therapy approaches (Mehraj et al., 2022). Despite these advances, challenges remain in translating these findings into clinical practice, necessitating further investigation into the complex interplay between TOP2A status and cancer biology.

This investigation found that HJURP is overexpressed in clinical samples of GC, with higher HJURP levels positively linked to cell cycle activity and DNA repair processes in GC. Knockdown of HJURP substantially suppressed cell growth and decreased chemoresistance in GC cells. The study also provided evidence that HJURP facilitates TOP2A transcription by improving MYC stability. Collectively, these findings suggest that HJURP may represent a potential therapeutic target for GC.

## 2 Material and methods

### 2.1 Patients and tissue samples

During the period from January to December 2023, six pairs of fresh GC tissues along with their matched adjacent normal tissues were collected from the Department of Gastrointestinal Surgery at the Affiliated Hospital of Nantong University. The tissues were stored in RNA later (ThermoFisher, catalog number AM7021). The main inclusion criteria were as follows:

1. Patients diagnosed with GC based on histopathological evaluation.
2. Patients without any concurrent malignant tumors or severe diseases affecting other organs.
3. Patients who had not received chemotherapy, radiotherapy, or immunotherapy prior to surgical intervention.
4. Patients with comprehensive clinical records.

Follow-up assessments were conducted both at the outpatient department and via telephone calls. The study was approved by the Human Research Ethics Committee of the Affiliated Hospital of Nantong University, and each participant provided signed informed consent (2023-K076-01).

### 2.2 Cell culture

The GC cell lines SNU-216 and MKN-45 were acquired from BeNa Culture Collection in Beijing, China. The AGS and HGC-27 GC cell lines were obtained from the National Collection of Authenticated Cell Cultures in Shanghai, China. Additionally, the BGC823 was gifted from Professor Q Guo, China Pharmaceutical University. All these cell lines were maintained in RPMI-1640 medium, which was enriched with 10% fetal bovine serum and antibiotics (100 U/mL penicillin and streptomycin). The fetal bovine serum was provided by Clark (Shanghai, China), while the antibiotics were supplied by Life Technologies (Shanghai, China). Cells were incubated at 37°C with 5% CO_2_.

### 2.3 RNA extraction and quantitative real-time PCR (qRT-PCR)

Total RNA was isolated using the RNA isolater Total RNA Extraction Reagent (Vazyme, Nanjing, product code R401-01). The extracted RNA was then reverse-transcribed into cDNA using the HiScript III RT SuperMix for qPCR (+gDNA wiper) kit (Vazyme, Nanjing, product code R23-01). Quantitative real-time PCR (qRT-PCR) was conducted with the ChamQ Universal SYBR qPCR Master Mix (Vazyme, Nanjing, product codes Q711-02/03) on an Applied Biosystems QuantStudio 5 instrument (Thermo Fisher Scientific, United States). The primer sequences utilized in this study are as follows:

HJURP forward 5′-GATTCAAAAAGCGGTGAGGTCG-3′

HJURP reverse 5′-AGTCACACGTACATCCCTTCC-3′

TOP2A forward 5′-ACCATTGCAGCCTGTAAATGA-3′

TOP2A reverse 5′-GGGCGGAGCAAAATATGTTCC-3′

GAPDH forward 5′-CTTAGCACCCCTGGCCAAG-3′

GAPDH reverse: 5′- GATGTTCTGGAGAGCCCCG-3′

### 2.4 Western blot analysis

Western blot analysis was performed according to previously established methods (Hu et al., 2024; Wu et al., 2017; Ou et al., 2024b; Li et al., 2017). Protein extraction was conducted using Radioimmunoprecipitation Assay (RIPA) buffer from Epizyme (Shanghai, China). The protein concentration was measured using the Omni-Rapid™ Protein Quantitation Kit (Epizyme, Shanghai, China). The following antibodies were used: GAPDH (60004-1-Ig), HJURP (15283-1-AP), and TOP2A (66541-1-Ig) from Proteintech (Wuhan, China); Phospho-H2AX (bs-3185R) from Bioss (Beijing, China); and MYC (MAB3696) from R&D Systems (MN, United States).

### 2.5 Cell proliferation assays

The proliferative capacity of GC cells was assessed using 5-Ethynyl-2′-deoxyuridine (EdU) incorporation, Cell Counting Kit-8 (CCK-8), and colony formation assays. These assays were conducted following previously described protocols (Zhang Y. et al., 2021). To evaluate the chemosensitivity of GC cells, colony formation assays were specifically employed. In these sensitivity detection experiments, cells were seeded into 6-well plates and allowed to adhere for 24 h. Subsequently, they were treated with cisplatin (DDP, 1 μg/mL) for 2 h. Following this treatment, the conventional complete culture medium was replaced.

### 2.6 Bioinformatics analysis

The gene expression data for HJURP were obtained from the corrected The Cancer Genome Atlas (TCGA) dataset, specifically the RNA-seq data provided by the PanCanAtlas (https://www.cancer.gov/tcga). These data are sourced from the EBPlusPlusAdjustPANCAN_IlluminaHiSeq_RNASeqV2.geneExp.tsv file. To standardize the expression levels across different tumor types, the data were transformed into unitless Z-Score values. This transformation was performed using the formula (x - μ)/σ, where:

- x represents the expression value of the gene in a given sample,
- μ is the mean expression value of that gene across all samples of the same tumor type, and
- σ denotes the standard deviation of the gene’s expression across all samples of the same tumor type. Z-Score values less than −3 or greater than 3 are considered outliers and are removed from the dataset. Following outlier removal, only tumors with at least three corresponding normal samples are included in the analysis. Statistical differences in expression levels between tumor and normal tissues within the digestive system tumor datasets are compared using Wilcoxon Rank Sum Tests.

Paired differential gene expression analysis: (Same data as before) Using Wilcoxon Signed Rank Tests to compare the statistic difference of the expression level between the cancer/paracancer tissues in the TCGA dataset.

Gene Expression Omnibus (GEO) Data Analysis: The analysis workflow involves systematically downloading relevant GEO datasets and converting probe-level matrices into gene-level matrices, following the guidelines provided in the respective platform files for each dataset. For genes represented by multiple probes, the expression levels are averaged to ensure accurate representation. Next, the expression data are normalized to unitless Z-Score values for each tumor sample using the formula (x - μ)/σ, where:

- x is the gene expression value,
- μ is the mean expression value across samples of the same tumor type, and
- σ is the standard deviation of expression within the same tumor type.

To assess statistical differences in expression between tumor and normal tissues within the dataset, Wilcoxon Rank Sum Tests are employed.

Receiver Operating Characteristic (ROC) Curve Analysis: The pROC package is utilized to conduct ROC analysis, which includes calculating the total area under the curve (AUC) and the 95% confidence interval, as well as plotting a smooth ROC curve. This analysis aims to evaluate the diagnostic performance of gene expression in distinguishing between the tumor disease group and the normal group.

Analysis of subtype: Tumor samples are divided into high/low expression groups according to the median value, the proportion of each subtype in each group is calculated, and Chi-square test is used to detect significance.

Gene set Variation Analysis (GSVA): The CancerSEA database sorted out the different functional states of 14 tumor cells. The z-score algorithm was proposed by Yuan et al. (2019). The activity of a given pathway was reflected by integrating the expression of characteristic genes, using R package GSVA in z-score parameter calculated the 14 functional state gene sets and obtained the combined z-score. We use the scale function to further standardize the score as the gene set score, and calculate the Pearson correlation between the gene and each gene set score (Hanzelmann et al., 2013).

### 2.7 Small interfering RNA (siRNA) and overexpression vector experiments

Small interfering RNAs (siRNAs) targeting HJURP and TOP2A were obtained from Tsingke Biotech (Beijing, China). Approximately 1 × 10^5 cells per well were seeded in 6-well plates and allowed to adhere for 24 h. Following this incubation period, HJURP/TOP2A siRNAs were transfected into the cells using Polyplus jetPRIME transfection reagent (Strasbourg, France) according to the manufacturer’s protocol. Cells were harvested 48 h post-transfection for RNA and protein isolation, and knockdown efficiency was evaluated. For overexpression studies, the cDNA sequences of HJURP (NM_001282962.2) and TOP2A (NM_001067.4) were cloned into the pCMV3 vector provided by Nanjing Corues Biotech (Jiangsu, China). The resulting overexpression vectors were transfected into gastric cancer (GC) cells using Polyplus jetPRIME, following the manufacturer’s instructions (Ou et al., 2024b). All experiments were conducted independently in triplicate to ensure reproducibility.

### 2.8 Statistical analysis

During the analysis of experimental data, we initially used Excel software for preliminary processing and analysis of the obtained data. Subsequently, to more professionally illustrate trends and distributions in the data, we utilized GraphPad Prism 10.0 (GraphPad Software, Inc., San Diego, CA, United States) for graph plotting.

For comparisons among multiple groups, one-way analysis of variance (One-way ANOVA) was performed, followed by *post hoc* tests to further investigate group differences. For comparisons between two groups, Student’s t-test was utilized. Specifically, when comparing the means of two independent samples, an unpaired two-tailed Student’s t-test was applied. All experimental data were obtained from at least three independent replicate experiments to ensure reliability and reproducibility.

Survival outcomes were assessed using Kaplan-Meier curves, with statistical significance evaluated via the log-rank test and Cox regression analysis (Gonzalez et al., 2024). Statistical significance or P values are denoted as follows: *p < 0.05; **p < 0.01; ***p < 0.001; ****p < 0.0001; ns indicates no statistical significance. Error bars in figures represent the mean ± standard deviation (SD).

## 3 Results

### 3.1 Elevated expression of HJURP in GC and its association with cell cycle and DNA repair

**FIGURE 1:**
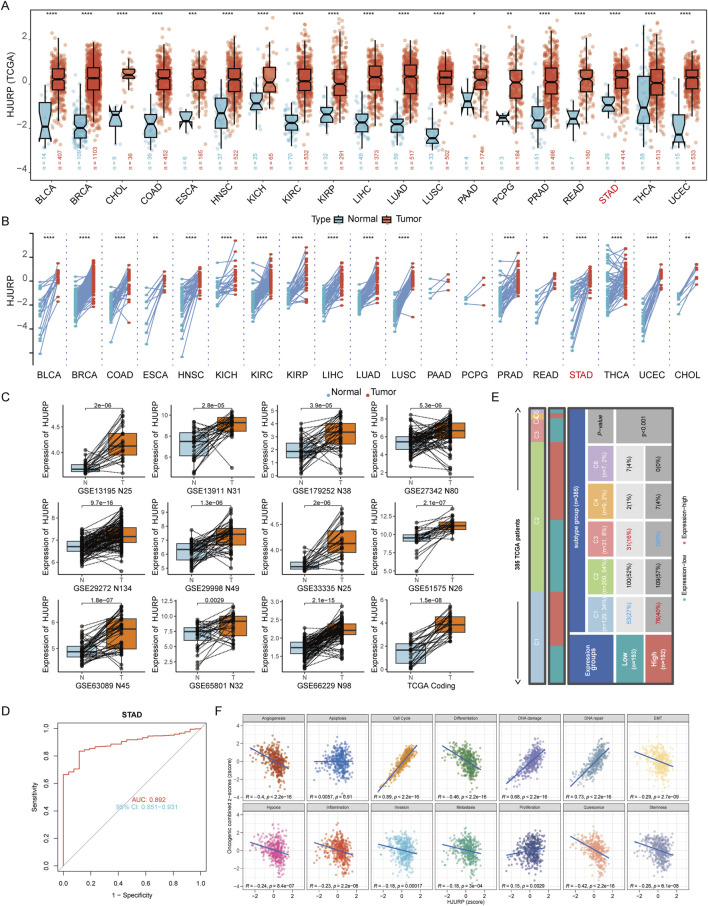
HJURP mRNA expression levels in GC. **(A, B)** Unpaired and paired analysis of HJURP mRNA expression level in normal tissues and cancers from TCGA databases. **(C)** HJURP mRNA expression level in normal and GC tissues from GEO database. **(D)** The ROC analysis of diagnostic accuracy for GC with HJURP expression in TCGA databases. **(E)** The identification of six distinct immune subtypes in GC patients. **(F)** GSVA showed the functional implications of HJURP expression in GC patients. *P < 0.05, **P < 0.01, ***P < 0.001, ****P < 0.0001. GC: gastric cancer, HJURP: Holliday Junction Recognition Protein, TCGA: The Cancer Genome Atlas, GEO: Gene Expression Omnibus, ROC: Receiver Operating Characteristic, GSVA: Gene set Variation Analysis.

We initially analyzed the expression levels of HJURP across a pan-cancer cohort using RNA-seq data from TCGA. Compared to adjacent normal tissues, HJURP expression was significantly upregulated in nearly all types of cancer tissues, regardless of whether the samples were paired or unpaired (Figures 1A, B). Next, we evaluated HJURP expression in GC tissues and their paired adjacent normal tissues using 11 datasets from the GEO and TCGA-STAD databases (Figure 1C). The results confirmed a significant upregulation of HJURP in GC tissues, consistent with the findings from the TCGA analysis. Further analysis using TCGA-STAD data revealed that HJURP expression showed high diagnostic accuracy for GC. ROC curve analysis indicated an AUC value of 0.892, with a 95% confidence interval ranging from 0.851 to 0.931 (Figure 1D).

According to the ‘Immune Landscape of Cancer' study, which conducted a large-scale immunogenomic analysis of over 10,000 tumor samples across 33 different cancer types in TCGA, six distinct immune subtypes were identified (Thorsson et al., 2018). Our analysis of GC data indicated that the C1 subtype (wound healing) was predominant in the high HJURP expression group, while the low HJURP expression group showed a significant increase in the proportion of the C3 subtype (inflammatory) (Figure 1E). To further explore the functional implications of HJURP expression, we performed GSVA. This analysis demonstrated that in GC, HJURP expression was positively correlated with scores related to cell cycle, DNA damage, DNA repair, and cell proliferation (Figure 1F). Both the immune subtype analysis and GSVA results suggest that HJURP expression is closely associated with cellular proliferative capacity.

### 3.2 Knockdown of HJURP reduces proliferation in GC cell lines

**FIGURE 2:**
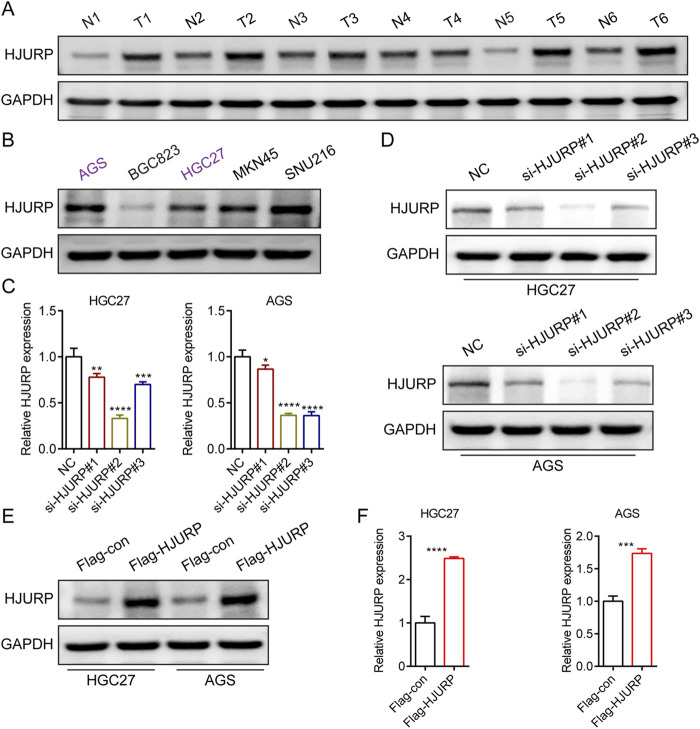
HJURP protein expression levels in GC cell lines and tissues. **(A)** Western blot showed the expression of HJURP in paired normal tissues and GC tissues. **(B)** Western blot showed the expression of HJURP in different GC cell lines. **(C, D)** HGC27 and AGS were transfected with NC siRNA or HJURP siRNA for 48 h and then cell samples were collected, and the levels of HJURP were determined by qRT-PCR and Western blot. **(E, F)** HGC27 and AGS were transfected with Flag-con or Flag-HJURP for 48 h and then cell samples were collected, and the levels of HJURP were determined by qRT-PCR and Western blot. *P < 0.05, **P < 0.01, ***P < 0.001, ****P < 0.0001. GC: gastric cancer, HJURP: Holliday Junction Recognition Protein.

To evaluate the expression profile of HJURP in tissues, we analyzed the protein levels of HJURP in paired cancerous and adjacent non-tumor tissues from 6 GC patients. Our results revealed a significantly higher expression of HJURP in tumor tissues compared to their corresponding non-tumor tissues (Figure 2A). We screened multiple GC cell lines for HJURP expression levels and chose HGC27 and AGS cells, which displayed moderate expression levels of HJURP, for subsequent experiments (Figure 2B). Subsequently, to validate the knockdown and overexpression efficiency of si-HJURP and Flag-HJURP in these 2 GC cell lines, we performed Western blot and qRT-PCR analyses. The results indicated that among the tested siRNA sequences, si-HJURP#2 achieved the most potent silencing effect on HJURP when compared to the negative control (NC) (Figures 2C, D). Moreover, the overexpression vector Flag-HJURP was efficiently expressed in both HGC27 and AGS cells, as evidenced by increased protein and mRNA levels (Figures 2E, F).

**FIGURE 3:**
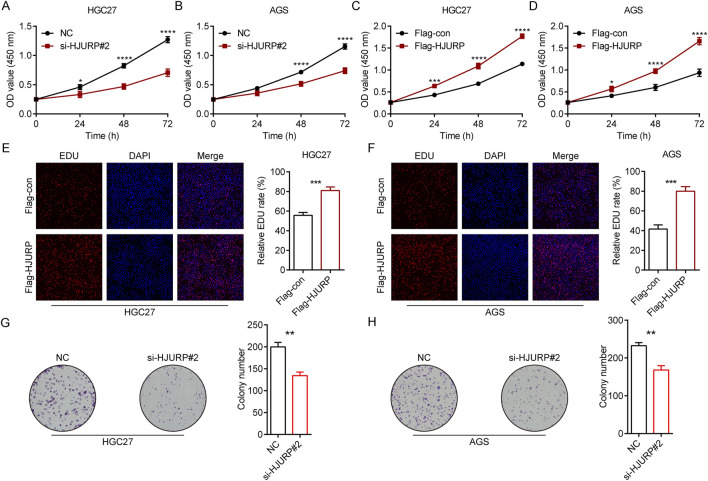
The effects of HJURP expression on the proliferation of GC cell lines. **(A)** CCK-8 assays were employed to evaluate the effect of HJURP knockdown on the proliferation of HGC27 GC cells. **(B)** CCK8 assays were employed to evaluate the effect of HJURP knockdown on the proliferation of AGS GC cells. **(C)** CCK8 assays were employed to evaluate the effect of HJURP overexpression on the proliferation of HGC27 GC cells. **(D)** CCK8 assays were employed to evaluate the effect of HJURP overexpression on the proliferation of AGS GC cells. **(E, F)** EdU assays were employed to evaluate the effect of HJURP overexpression on the proliferation of HGC27 and AGS GC cells. **(G, H)** Colony formation assays CCK8 assays were employed to evaluate the effect of HJURP knockdown on the proliferation of HGC27 and AGS GC cells. *P < 0.05, **P < 0.01, ***P < 0.001, ****P < 0.0001. GC: gastric cancer, HJURP: Holliday Junction Recognition Protein, CCK-8: Cell Counting Kit-8, EdU: 5-Ethynyl-2′-deoxyuridine.

To evaluate the role of HJURP in the growth of GC cells, we employed CCK-8 assays, colony formation assays, and EdU assays. The results demonstrated that the knockdown of HJURP using si-HJURP#2 significantly reduced the OD values in both HGC27 (Figure 3A) and AGS (Figure 3B) cell lines compared to the NC. Conversely, overexpression of HJURP using the Flag-HJURP plasmid led to a significant increase in OD values in both HGC27 (Figure 3C) and AGS (Figure 3D) cell lines compared to the Flag-con group. The EdU assay revealed a significant increase in the percentage of EdU-positive cells in both HGC27 (Figure 3E) and AGS (Figure 3F) cell lines upon HJURP overexpression. Moreover, the number of colonies formed by these cells was significantly decreased following HJURP knockdown (Figures 3G, H). These results indicated that HJURP positively regulates the proliferation of GC cells. Specifically, the reduction in HJURP expression led to decreased cell viability and proliferation, while increased HJURP expression enhanced these parameters, suggesting a critical role for HJURP in the growth and proliferation of GC cells.

### 3.3 HJURP overexpression enhances chemoresistance in GC cells

**FIGURE 4:**
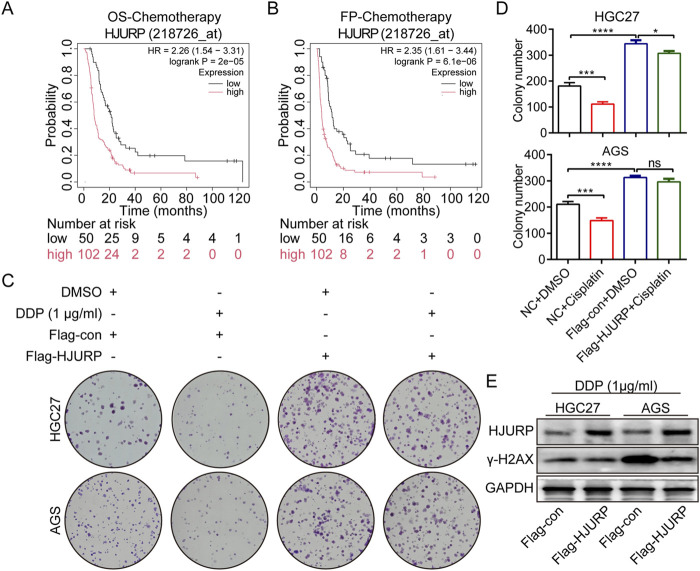
The effects of HJURP expression on the chemoresistance of GC cell lines. **(A)** The relationship between the expression of HJURP in Kaplan–Meier Plotter database and OS in GC patients. **(B)** The relationship between the expression of HJURP in Kaplan–Meier Plotter database and FP in GC patients. **(C, D)** Colony formation assays were employed to evaluate the effect of HJURP overexpression on the chemoresistance of HGC27 and AGS. **(E)** Western blot showed the γ-H2AX in HGC27 and AGS transfected with Flag-HJURP followed with DDP (1 μg/mL). *P < 0.05, ***P < 0.001, ****P < 0.0001. GC: gastric cancer, HJURP: Holliday Junction Recognition Protein, OS: overall survival, FP: first progression survival, DDP: cisplatin.

To investigate the impact of HJURP expression on GC patient survival, we analyzed data from the KM plotter database (http://www.kmplot.com/). Our findings indicated that while high versus low HJURP expression did not significantly affect overall survival (OS), it was negatively correlated with first progression survival (FP) in GC patients (Supplementary Figures S1A, B). We further stratified GC patients based on their treatment modalities into three groups: Surgery Only, Chemotherapy, and Other Adjuvant therapies. Notably, in the Chemotherapy group, higher HJURP expression was significantly associated with shorter OS and FP (Figures 4A, B, Supplementary Figures S1A, B). This observation aligns with our earlier finding that HJURP is positively correlated with DNA damage repair (Figure 1F), suggesting a potential link between HJURP expression and chemotherapeutic drug sensitivity.

To investigate the role of HJURP in chemoresistance, we conducted additional experiments. We transfected GC cells with the Flag-HJURP overexpression vector and then treated them with cisplatin (DDP). The results demonstrated that HJURP-overexpressing cells exhibited enhanced resistance to DDP compared to control cells, as evidenced by increased cell viability and reduced apoptosis rates (Figures 4C, D). Western blot results demonstrated that DDP-induced γ-H2AX protein expression, which serves as a marker for DNA damage, was markedly diminished in cells overexpressing HJURP (Figure 4E). This reduction in γ-H2AX levels implies a lower extent of DNA damage in HJURP-overexpressing cells. Collectively, these observations suggest that increased HJURP expression may contribute to chemoresistance in GC by promoting more efficient DNA damage repair. This finding underscores the significance of HJURP as a potential therapeutic target, especially in strategies aimed at enhancing chemosensitivity in GC treatment.

### 3.4 HJURP upregulates the mRNA levels of TOP2A

**FIGURE 5:**
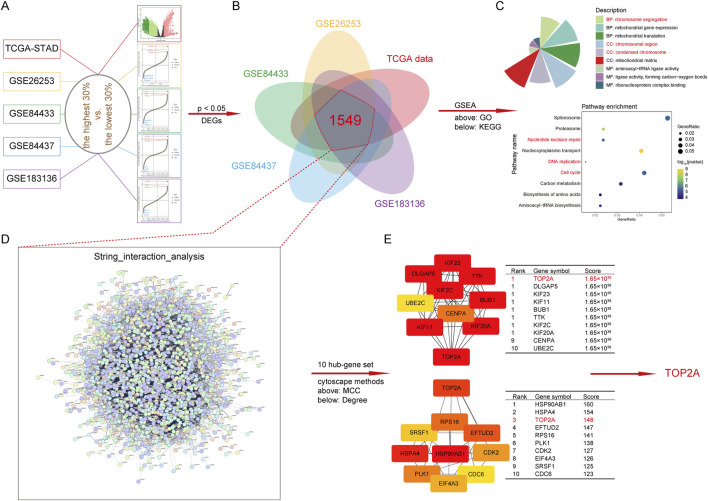
Functional enrichment analysis and downstream regulatory gene of HJURP. **(A)** Volcanic plot of HJURP differentially expressed genes (DEGs) in TCGA-STAD and GEO (GSE26253, GSE84433, GSE84437, and GSE183136) datasets. **(B)** The Venn diagram displays the intersection results of DEGs in TCGA-STAD and GEO (GSE26253, GSE84433, GSE84437, and GSE183136) datasets. **(C)** Chart of GO and Bubble plot of KEGG enrichment pathways based on DEGs. **(D)** The interaction analysis of DEGs using String dataset (cn.string-db.org/). **(E)** The determination of hub gene by Cytoscape (https://cytoscape.org/) with MCC and Degree methods. DEGs: differentially expressed genes, TCGA: The Cancer Genome Atlas, GEO: Gene Expression Omnibus, GO: Gene Ontology, KEGG: Kyoto Encyclopedia of Genes and Genomes, MCC: Maximal Clique Centrality, TOP2A: Topoisomerase II alpha.

To reveal the complex mechanism by which HJURP induces GC cell progression, we embarked on an exploration using TCGA-STAD and GEO (GSE26253, GSE84433, GSE84437 and GSE183136) databases with sample sizes exceeding 100. Firstly, we extracted tumor samples from a specific dataset and divided them into two groups based on a cutoff value of 0.3: the top 30% of samples with the highest HJURP expression and the bottom 30% with the lowest HJURP expression. We performed differential expression analysis using the limma package. We visualized the top genes using a volcano plot or a waterfall plot (Figure 5A). Genes with an adjusted p-value <0.05 were considered significantly differentially expressed. We then identified the intersection of differentially expressed genes (DEGs) across five distinct datasets, yielding a list of 1,549 candidate genes (Figure 5B). To further understand the biological significance of these DEGs, we conducted Gene Ontology (GO) and Kyoto Encyclopedia of Genes and Genomes (KEGG) enrichment analyses. These analyses revealed significant enrichment in pathways related to chromatin stability, cell cycle regulation, and DNA damage repair (Figure 5C). Among the 1,549 DEGs, how does HJURP exert its regulatory functions? Hub genes play pivotal roles in biological processes, often acting as master regulators within pathways and serving as important therapeutic targets and research foci. Therefore, we analyzed these 1,549 genes using the STRING database to identify potential hub genes. By applying Maximal Clique Centrality (MCC) and Degree methods, our analysis identified TOP2A as a key hub gene, with significance in both methods (Figures 5D, E). Thus, we selected TOP2A for further exploration.

### 3.5 HJURP improves transcriptional activity of TOP2A in GC cells

**FIGURE 6:**
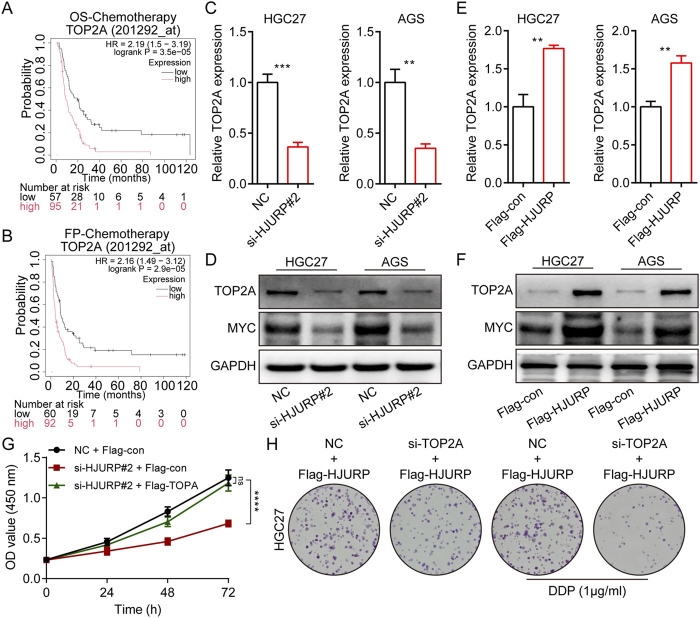
HJURP promotes proliferation and chemoresistance of GC cell by activating the TOP2A. **(A)** The relationship between the expression of TOP2A in Kaplan–Meier Plotter database and OS in GC patients. **(B)** The relationship between the expression of TOP2A in Kaplan–Meier Plotter database and FP in GC patients. **(C, D)** HGC27 and AGS were transfected with NC siRNA or HJURP siRNA for 48 h and then cell samples were collected, and the levels of TOP2A were determined by qRT-PCR and Western blot. **(E, F)** HGC27 and AGS were transfected with Flag-con or Flag-HJURP for 48 h and then cell samples were collected, and the levels of TOP2A were determined by qRT-PCR and Western blot. **(G)** CCK-8 assays were employed to evaluate the effect of HJURP knockdown with or without TOP2A overexpression on the proliferation of HGC27 GC cells. **(H)** Colony formation assays were employed to evaluate the effect of HJURP overexpression with or without TOP2A knockdown on the chemoresistance of HGC27 and AGS. **P < 0.01, ***P < 0.001, ****P < 0.0001. GC: gastric cancer, HJURP: Holliday Junction Recognition Protein, OS: overall survival, FP: first progression survival, DDP: cisplatin, TOP2A: Topoisomerase II alpha.

Kaplan-Meier survival analysis revealed that among patients receiving chemotherapy, those with high TOP2A expression had significantly shorter overall survival (OS) and first progression (FP) times (Figures 6A, B). In GC cell lines, we conducted RT-qPCR and Western blot analyses following HJURP knockdown. The results showed a marked decrease in TOP2A mRNA and protein levels when HJURP was downregulated (Figures 6C, D). On the other hand, overexpression of HJURP led to a notable increase in TOP2A expression at both the mRNA and protein levels (Figures 6E, F). Cell rescue experiments confirmed that re-expression of TOP2A in HJURP-knockdown cells restored the diminished proliferative capacity (Figure 6G). Similarly, overexpression of HJURP in HGC27 cells markedly reduced cisplatin sensitivity, while simultaneous knockdown of TOP2A significantly enhanced cisplatin sensitivity (Figure 6H). These findings indicate that HJURP transcriptionally activates TOP2A (Figures 6C, E).

**FIGURE 7:**
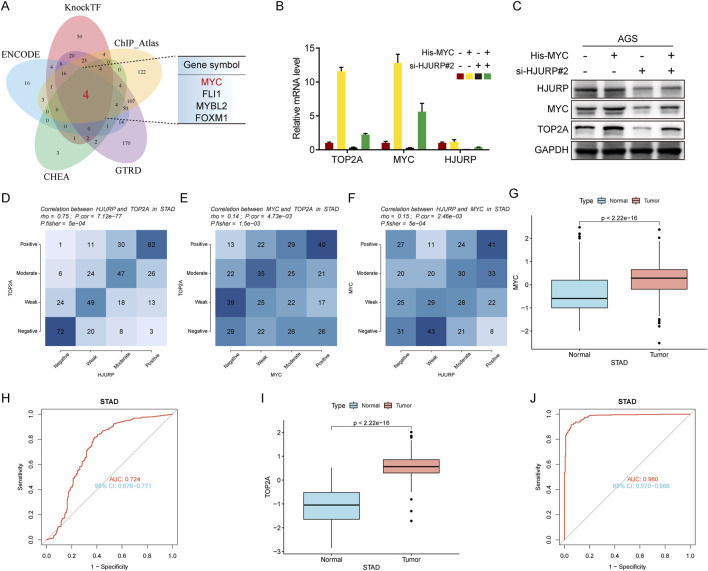
HJURP improves transcriptional activity of TOP2A via the promotion of MYC signaling. **(A)** The Venn diagram displays the potential transcription factors of TOP2A. **(B, C)** AGS were transfected with HJURP siRNA or His-MYC for 48 h and then cell samples were collected, and the levels of TOP2A, MYC and HJURP were determined by qRT-PCR and Western blot. **(D–F)** Genes are divided into 4 classes (Positive, Moderate, Weak, Negative) according to the expression level (visualized by contingency table heat map, and the depth of color represents the number of samples. The Pearson correlation of the two genes is calculated and Fisher’s exact test is performed. **(G)** The difference of MYC expression in TCGA-STAD dataset. **(H)** The ROC analysis of diagnostic accuracy for GC with MYC expression in TCGA databases. **(I)** The difference of TOP2A expression in TCGA-STAD dataset. **(J)** The ROC analysis of diagnostic accuracy for GC with TOP2A expression in TCGA databases. TCGA: The Cancer Genome Atlas, HJURP: Holliday Junction Recognition Protein, TOP2A: Topoisomerase II alpha.

To further elucidate the transcription factors involved, bioinformatics predictions identified MYC, FLI1, MYBL2, and FOXM1 as potential regulators of TOP2A across multiple databases (Figure 7A). Given previous evidence that HJURP promotes malignant progression and mediates cisplatin sensitivity via MYC in serous ovarian cancer, we focused on MYC for further investigation (Dou et al., 2022). Consistent with prior research, altering HJURP expression in HGC27 and AGS cell lines led to notable changes in MYC protein levels, confirming the positive regulatory relationship between HJURP and MYC (Figure 6D). Further investigation demonstrated that knockdown of HJURP in AGS cells markedly reduced both mRNA and protein levels of MYC and TOP2A. Notably, overexpression of MYC via plasmid transfection exclusively restored the expression of MYC and TOP2A at transcriptional and translational levels, without affecting HJURP expression. Notably, when HJURP-targeting siRNA was co-transfected with the MYC-overexpressing plasmid, HJURP expression remained markedly suppressed, whereas the diminished expression of both MYC and TOP2A was partially rescued (Figures 7B, C). These findings strongly support a regulatory hierarchy in which HJURP modulates the transcriptional activity of MYC, thereby influencing downstream TOP2A expression. Subsequent analysis of TCGA data revealed significant positive correlations between the mRNA levels of TOP2A and HJURP, TOP2A and MYC, as well as MYC and HJURP (Figures 7D–F). Moreover, TCGA-STAD data indicated that both MYC and TOP2A were markedly upregulated in tumor tissues compared to adjacent normal tissues. These genes also showed high diagnostic accuracy for gastric cancer, as evidenced by their performance metrics (Figures 7G–J).

## 4 Discussion

Emerging evidence indicates that HJURP is frequently overexpressed in various types of cancer and plays a crucial oncogenic role in cancer progression (Dunleavy et al., 2009; Kato et al., 2007). Hypomethylation-driven overexpression of HJURP promotes the progression of hepatocellular carcinoma (HCC) and is associated with poor prognosis (Li et al., 2021). Specifically, HJURP enhances HCC proliferation by destabilizing p21 through the MAPK/ERK1/2 and AKT/GSK3β signaling pathways (Chen et al., 2018). In non-small cell lung cancer (NSCLC), knocking down HJURP inhibits cell proliferation, migration, and invasion by repressing Wnt/β-catenin signaling (Wei et al., 2019). Additionally, modulation of HJURP levels has been linked to glioblastoma cell survival (Valente et al., 2013). Despite these findings, the role of HJURP in GC remains incompletely understood. Consistent with the aforementioned studies, our research demonstrates that HJURP is upregulated in both GC cell lines and tissues, and its overexpression is associated with malignant characteristics. This suggests that HJURP may play a functional role in driving GC. Our study found that HJURP promotes GC growth and enhances chemotherapy resistance *in vitro*, providing further support for the hypothesis that HJURP is a functional driver of GC. These findings highlight the potential of HJURP as a therapeutic target and biomarker in GC treatment. Recent advances in liquid biopsies, circulating tumor DNA (ctDNA) testing, and methylation signatures have transformed cancer diagnosis and monitoring (Jahangiri, 2024; Ohyama et al., 2024; Gonzalez et al., 2024). These techniques are powerful tools for detecting genetic and epigenetic changes, and the applicability of the transcriptional regulatory networks involved in HJURP requires further investigation.

Numerous studies have provided evidence that growth and chemoradiotherapy resistance represent key processes in the development of malignant tumor (Joshi and Badgwell, 2021; Ouyang et al., 2022; Yang et al., 2024; Yu et al., 2023). Owing to the absence of typical early clinical symptoms and effective screening methods, the majority of GC patients are diagnosed at an advanced stage, resulting in poor prognoses (Smyth et al., 2020). As a result, chemoradiotherapy assumes a critical role in the management of advanced GC (Joshi and Badgwell, 2021; Leong et al., 2024; Zhang H. et al., 2025). However, primary or acquired resistance to therapy and the associated toxic side effects are major challenges that lead to treatment failure and a decline in quality of life, presenting significant hurdles during the course of treatment (Liu and Da, 2023). Previous research has established the involvement of HJURP in DNA repair and genomic stability. Recent studies have provided substantial evidence that HJURP and CENP-A play a crucial role in licensing homologous recombination (HR) during the G1 phase to repair Cas9-induced centromeric double-strand breaks (DSBs), even in the absence of sister chromatids (Jaramillo-Lambert et al., 2013). Misregulation of Scm3p/HJURP leads to chromosome instability in both *Saccharomyces cerevisiae* and human cells (Mishra et al., 2011). HJURP functions as a CENP-A chromatin assembly factor, sufficient to form a functional *de novo* kinetochore (Barnhart et al., 2011). The expression level of HJURP has been shown to have an independent prognostic impact and can predict sensitivity to radiotherapy in breast cancer (Hu et al., 2010). HJURP binds to CENP-A via a highly conserved N-terminal domain, facilitating its deposition at centromeres (Shuaib et al., 2010). It acts as a cell-cycle-dependent maintenance and deposition factor for CENP-A at centromeres (Dunleavy et al., 2009), mediating the centromere-specific assembly of CENP-A nucleosomes (Foltz et al., 2009). Prior studies have demonstrated that HJURP promotes tumor cell proliferation and chemoradiotherapy resistance by participating in cell cycle regulation and maintaining genomic stability. In our current study, we conducted bioinformatics analysis of TCGA and GEO datasets for gastric cancer (GC) to identify differentially expressed genes (DEGs). Among these genes, pathways related to the cell cycle and nucleotide excision repair were significantly enriched. Further analysis revealed that TOP2A is a hub gene within the regulatory network of HJURP. Mechanistically, we found that HJURP increases the transcription level of TOP2A in GC cells. The TCGA has been instrumental in identifying biomarkers and understanding the molecular landscape of cancers. Studies such as those on SCN3B in glioma (Liu H. et al., 2024), CDK2 in glioma (Liu and Weng, 2022), AIMP1 in head-neck squamous cell carcinoma (Li and Liu, 2022) and CNIH4 in head and neck squamous cell carcinoma (Liu and Li, 2022) demonstrate the diverse applications of TCGA data in cancer research. However, TCGA studies are not without limitations. Technical and biological biases, such as those discussed in “Technical and Biological Biases in Bulk Transcriptomic Data Mining for Cancer Research” (Liu et al., 2025), can influence the reliability of findings. While TCGA provides a wealth of data for hypothesis generation, independent validation and multi-center studies are essential to address these biases and enhance the translational potential of TCGA discoveries.

TOP2A (DNA topoisomerase II alpha) is a critical enzyme that controls DNA supercoiling and is intimately involved in DNA replication, transcription, and chromatin remodeling (Nitiss, 2009; Zou et al., 2024; Lovejoy et al., 2025). Extensive research has highlighted the importance of TOP2A in cancer cell proliferation and its potential as a therapeutic target (Delgado et al., 2018). Overexpression of TOP2A is observed in multiple types of cancer, such as lung, breast, and liver cancer, and it is linked to tumor progression, chemoresistance, and resistance to radiotherapy (Sritharan and Sivalingam, 2021; Glisson and Ross, 1987; Chen et al., 2024; Jiang et al., 2024). Specifically, in the context of tumor cell proliferation, TOP2A is especially crucial during the S and G2/M phases of the cell cycle (Deng et al., 2023). During these phases, TOP2A mitigates the topological stress that occurs during DNA replication, ensuring smooth progression through the cell cycle and supporting efficient cell division. This makes it an attractive target for anticancer drugs, many of which inhibit TOP2A and induce DNA damage, leading to cell death. Research into the mechanisms of chemoresistance mediated by TOP2A has revealed that its overexpression can lead to decreased susceptibility to chemotherapeutic agents that target the enzyme (Zhu et al., 2021). For instance, in breast cancer, O-GlcNAcylation of TOP2A has been shown to enhance its catalytic activity, which in turn contributes to chemoresistance (Liu et al., 2023). This post-translational modification stabilizes the enzyme-DNA cleavage complex, reducing the effectiveness of drugs like doxorubicin. In terms of radiotherapy, TOP2A has been implicated in the development of radio-resistance in prostate cancer (Hansen et al., 2023). Moreover, TOP2A’s role in non-small cell lung cancer (NSCLC) has been investigated in relation to immunotherapy and vasculogenic mimicry (Wu et al., 2023). Studies suggest that TOP2A may promote the expression of immune checkpoint molecules like PD-L1 and the formation of vasculogenic mimicry, which are associated with poor prognosis and resistance to therapy. The current landscape of research indicates that TOP2A is a multifaceted factor in tumorigenesis and treatment resistance. In agreement with this hypothesis, our research shows that HJURP overexpression boosts the transcriptional activity of TOP2A through the upregulation of MYC. This cascade of events leads to enhanced genomic stability, which facilitates GC cell proliferation and confers resistance to chemoradiotherapy.

MYC is a crucial transcription factor closely associated with various biological processes, including cell proliferation, metabolism, and apoptosis. There are reports indicating that excessive MYC induces DNA damage by increasing transcriptional stress (Vafa et al., 2002; Das et al., 2024). For instance, excessive MYC activity has been shown to trigger DNA damage responses, activating pathways such as p53, which attempts to halt cell cycle progression and initiate repair mechanisms. The activation of p53 in response to MYC-induced stress is a protective mechanism that attempts to mitigate the DNA damage caused by MYC’s transcriptional activity (Das et al., 2024). However, our research results confirmed that HJURP reduces apoptosis and DNA damage and positively regulates MYC expression. Actually, studies have highlighted that MYC not only plays a significant role in tumor development but also exhibits a dual role in the induction of DNA damage. The dual effects of MYC have also been demonstrated in other types of cancer. In hepatocellular carcinoma (HCC), overexpression of MYC is closely related to cell cycle progression and apoptosis, and inhibition of MYC can significantly improve the sensitivity of HCC cells to chemotherapy (Papadopoulos et al., 2023). In addition, MYC also influences cell response to DNA damage by regulating gene expression related to DNA damage repair. Studies have found that there is a complex interaction between MYC and DNA damage repair network, which provides a new perspective for developing new cancer treatment strategies (Kaller et al., 2023). It is important to note that the relationship between MYC and DNA damage is complex and context-dependent. While previous studies have shown that excessive MYC can induce DNA damage through increased transcriptional stress, the role of MYC in our experimental system may be different due to the involvement of HJURP. HJURP might fine tune the function of MYC, and the level of MYC upregulated by HJURP may not reach the threshold that causes significant transcriptional stress and subsequent DNA damage. HJURP may interact with a series of proteins involved in DNA repair pathways (Serafim et al., 2024; Kato et al., 2007; Sousa et al., 2019). This interaction may enhance the cell’s ability to repair DNA damage, even in the presence of increased MYC expression. The cells in our study may have a more efficient DNA repair mechanism, which can counteract the potential DNA - damaging effects of MYC. In conclusion, the dual role of MYC in DNA damage induction provides a new perspective for understanding the genesis and progression of tumors. MYC is not only a proto-oncogene that promotes cell proliferation, but also a key factor in maintaining genome stability. Future studies are needed to further explore the mechanisms of action of MYC in different cancer types in order to provide new targets and strategies for cancer treatment.

Collectively, our study revealed that HJURP strengthens TOP2A mRNA expression through enhancing the transcriptional activation ability of MYC. HJURP-MYC-TOP2A axis induces GC proliferation and chemoresistance. Therefore, the HJURP-MYC-TOP2A axis could serve as a potential target for the therapeutic of GC.

## Data Availability

The original contributions presented in the study are included in the article/Supplementary Material, further inquiries can be directed to the corresponding authors.

## Supplementary material

SUPPLEMENTARY FIGURE S1 The relationship between HJURP and the prognosis of STAD. **(A)** The relationship between the expression of HJURP in Kaplan–Meier Plotter database and OS in GC patients. **(B)** The relationship between the expression of HJURP in Kaplan–Meier Plotter database and FP in GC patients.
