# Supplementary figures and images for: HJURP modulates cell proliferation and chemoresistance via the MYC/TOP2A transcriptional axis in gastric cancer
Source: Front Mol Biosci. 2025 Apr 11;12:1566293. doi: 10.3389/fmolb.2025.1566293 (PMC12021643; doi:10.3389/fmolb.2025.1566293)

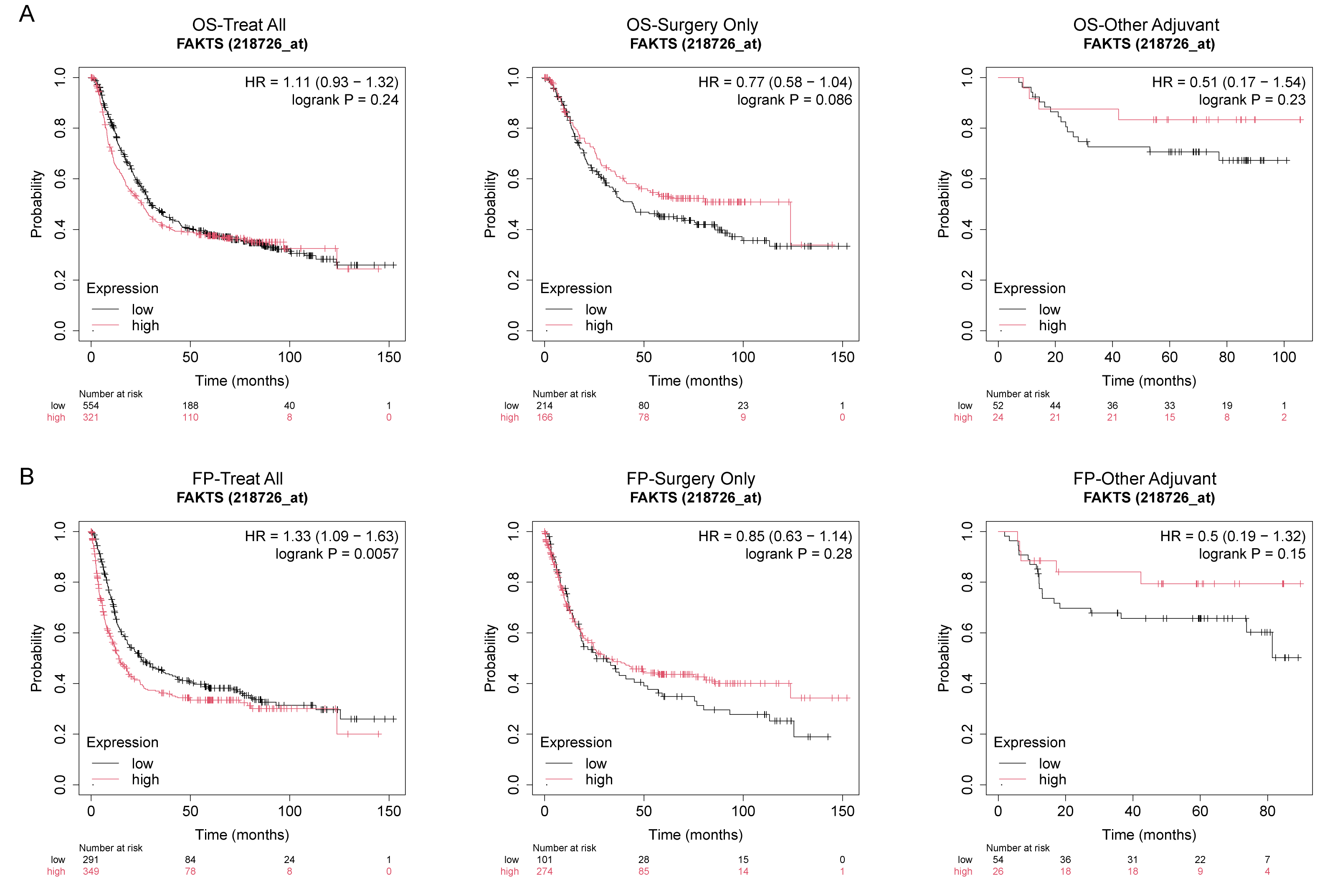

Supplement: Supplementary file 1 [file Image1.tif]
